# Prionoid Proteins in the Pathogenesis of Neurodegenerative Diseases

**DOI:** 10.3389/fnmol.2019.00271

**Published:** 2019-11-12

**Authors:** Cameron Wells, Samuel E. Brennan, Matt Keon, Nitin K. Saksena

**Affiliations:** Iggy Get Out, Neurodegenerative Section, Darlinghurst, NSW, Australia

**Keywords:** prion, prionoid, neurodegenerative disease, Alzheimer’s disease, Parkinsion’s disease, amyotrophic lateral scelerosis, Huntington’s disease

## Abstract

There is a growing body of evidence that prionoid protein behaviors are a core element of neurodegenerative diseases (NDs) that afflict humans. Common elements in pathogenesis, pathological effects and protein-level behaviors exist between Alzheimer’s Disease (AD), Parkinson’s Disease (PD), Huntington’s Disease (HD) and Amyotrophic Lateral Sclerosis (ALS). These extend beyond the affected neurons to glial cells and processes. This results in a complicated system of disease progression, which often takes advantage of protective processes to promote the propagation of pathological protein aggregates. This review article provides a current snapshot of knowledge on these proteins and their intrinsic role in the pathogenesis and disease progression seen across NDs.

## Introduction

Neurodegenerative diseases (NDs) are a range of debilitating conditions, predominantly involving the gradual loss of function and death of cells in the central and peripheral nervous system. Most neurons are incapable of reproducing to replace lost cells, so this damage is often cumulative and permanent. Prionoid disorders are a class of NDs characterized by protein aggregates which propagate *via* template-directed misfolding. Many prominent NDs are now believed to have a basis in prionoid pathology, including Alzheimer’s Diseases (AD), Parkinson’s Disease (PD), Huntington’s Disease (HD) and Amyotrophic Lateral Sclerosis (ALS). Although these diseases express varied etiology and pathology, each demonstrates the degeneration of neurons and gradual loss of neural functions. However, the specific neurons targeted and functions lost vary between diseases. Despite extensive research, our understanding of prionoid disease processes remains limited, and as of today, we have few effective treatments and no cures.

The study of prionoid disorders began with the identification of prions by Prusiner (1982), following decades of study into what had been previously referred to as “slow viruses” (Prusiner, 1982). His research brought several such diseases to the fore, beginning with Scrapie in sheep and later progressing to human prion protein (PrP). He identified prions as a self-templating amyloidogenic state of a normal cellular protein which served as an infectious pathogenic agent (Prusiner, 1994). These represented a novel form of pathology defined not solely by their genetic code, but by the abnormal conformations they take on and confer upon normally (“natively”) folded proteins.

The distinction between prions and prionoid proteins has long been a subject of academic discussion. Arguments have been made for various classification and naming conventions, yet no formal conclusion has been reached (discussed in Harbi and Harrison, 2014; Eraña, 2019). In this article, we will use the term “prionoid” to refer to proteins that display prionoid altered states in which they are capable of template-based self-replication and propagation between cells, but which have not demonstrated transmission between individuals.

In this review article, we summarize the current knowledge on prionoid protein disorders. We highlight the underlying mechanisms by which their intercellular transfer is mediated, resulting in pathologic neurodegenerative changes, as well as several proteins involved in neurodegenerative prionoid pathologies. We will specifically explore prionoid mechanisms in the pathologies and pathogenesis of AD (amyloid precursor protein, APP and Tau), HD (Huntingtin, Htt), PD (α-synuclein), and ALS [Fused in Sarcoma (FUS), Superoxide Dismutase 1 (SOD1), and TAR DNA-Binding Protein 43 (TDP-43)].

## Prionoid Proteins

Prionoid proteins are defined by their ability to misfold into at least one pathological conformation which can be transmitted to native forms of the protein. This templating function has been proposed to be facilitated by the exposure of hydrophobic amino acid side chains that are normally buried in the interior of the protein (Prusiner, 1998; Prusiner et al., 1998). Misfolded prionoid proteins share structures rich in β-sheets, polypeptide structures which render the proteins prone to forming aggregates comprised of protein fibrils (Cushman et al., 2010). These facilitate the development of intracellular aggregates that develop into stable inclusion bodies through the recruitment of native proteins. At the same time, extracellular fibrils can enter and seed aggregation in other cells, enabling intercellular transmissibility.

The specific mechanisms of activity vary between disorders, but they all ultimately lead to the death of a specific set of neurons in the brain. Disentangling the symptoms resulting from loss- or gain-of-function is often difficult, as the major function gained (prionoid protein misfolding) is often accompanied by loss of function (Allison et al., 2017). Leighton and Allison (2016) recently reviewed gain and loss of function mechanisms in AD, HD and ALS. In general, gain-of-function mechanisms include autophagic activation, aggregation, axonal dysfunction, and cellular stress, while loss-of-function entails protein sequestration, synaptic dysfunction and DNA damage. Some symptoms could be the result of either loss- or gain-of-function, such as denervation, mitochondrial dysfunction, excitotoxicity, and oxidative stress.

Immune responses such as the activation of glial cells are an early factor in many prionoid diseases and remain activated for an extended period of time (Sapp et al., 2001; Iannaccone et al., 2012; Liao et al., 2012; Kim et al., 2018). However, these processes are often ineffective, and prionoid proteins linger despite the ongoing activity of autophagic pathways. In the long term, this can result in a shift from a neuroprotective to a persistent neurotoxic environment. This serves to both impede pathological aggregate clearance and to exert neurotoxic effects (Liddelow et al., 2017). It appears likely that the disruption of the neuroprotective-neurotoxic equilibrium through extended immune activation plays a key role in the pathological effects of prionoid disorders.

## Prionoid Neurodegenerative Disorders Afflicting Humans

### Alzheimer’s Disease (AD)

AD is a neurodegenerative disorder characterized by progressive dementia, developing from subtle memory loss to severe memory loss and behavioral changes. This is caused by the progressive loss of synapses followed by the loss of neurons, particularly in regions of the brain involved in memory. These include the hippocampus, amygdala, entorhinal cortex, parahippocampal region, temporal pole and temporal lobe (Ramos Bernardes da Silva Filho et al., 2017). Most cases are sporadic (cause unknown), although there are rare inherited forms that result from mutations. These mutations are typically associated with the gene encoding APP or in presenilin 1 or 2, enzymes that contribute to the cleaving of APP into Aβ, both of which increase production of Aβ (Selkoe, 2001). The other significant genetic factor is apolipoprotein E (ApoE), with the ɛ4 and ɛ2 alleles increasing and decreasing AD risk respectively (Liu et al., 2013).

There are two major aggregates of prionoid proteins implicated in AD. These are extracellular neuritic plaques composed of amyloid-β (Aβ) peptides and intracellular neurofibrillary tangles (NFTs), cytoplasmic inclusion bodies rich in a hyperphosphorylated form of the tau protein.

### Amyloid-β

Aβ is a small peptide of 39–42 amino acids in length that is ubiquitously expressed in the central nervous system (CNS). It is formed by the cutting of APP by the enzymes β-secretase (BACE1) and γ-secretase in the membranes of neurons. While not well understood, APP and its isoforms have a broad array of functional roles outside of pathology, including involvement in synaptic plasticity and synaptogenesis, learning and memory, gene transcription and neuroprotection (reviewed in Hiltunen et al., 2009).

Aβ fibrils are toxic to mature neurons at high concentrations, causing dendritic and axonal retraction followed by cell death (Yankner et al., 1990). Aβ fibrils are rapidly cleared from the brains of healthy individuals, with a half-life between 1 and 2.5 h (Savage et al., 1998). However, this clearance is impeded in AD, with Aβ aggregates demonstrating the ability to impair the activity of the ubiquitin-proteasome system (Almeida et al., 2006; Mawuenyega et al., 2010). Varying levels of neurotoxicity are expressed by conformationally different forms of Aβ (Petkova et al., 2005). While Aβ40 (the peptide form ending at position 40 of APP) is drastically more abundant in the CSF, Aβ42 is either the major or sole component of neuritic plaques (Miller et al., 1993; Iwatsubo et al., 1994; Gravina et al., 1995). Overexpression of Aβ40 alone in transgenic mice does not lead to the formation of insoluble aggregates, and mixed aggregates develop more slowly than those containing Aβ42 alone (McGowan et al., 2005; Lei and Zhefeng, 2013). As the Aβ42 variant is significantly more toxic and prone to the formation of amyloid fibrils, this abundance is likely to play a role in AD pathogenesis (Phillips, 2019).

However, it is unlikely that neuritic plaques alone cause AD pathology. Multiple studies have shown a lack of direct correlation between plaque numbers and locations and AD-related damage (Arriagada et al., 1992; Gómez-Isla et al., 1997). He et al. (2018) recently proposed a model of AD pathology in which Aβ is necessary, but not itself a driver of pathological mechanisms. Instead, it creates an environment that promotes seeded tau pathology, and facilitates the accumulation of endogenous tau within nearby dystrophic axons. This increases fibrillization, which becomes a source of secondary tau seeds which can translocate *via* axons and spread pathology through iterative cycles of tau amplification.

A recent study into somatic recombination of APP identified a multitude of sequence variants of this gene, some of which were associated with AD pathogenesis. This study also revealed that AD brains contain three to five times more genetic variants than normal brains (Lee et al., 2018). This increase in variability may contribute to the variety of Aβ isoforms, inhibiting therapeutic activities targeted at only a portion of the Aβ population. Furthermore, it may result in proteins that are significantly more likely to aggregate and contribute to prionoid pathology. This may also contribute to the age-associated increase in AD, as the number of somatic genomic variants likewise increases with age.

#### Tau

Tau is a natively unfolded, soluble phosphoprotein. It is a major microtubule-associated protein (MAP) in mature neurons, interacting with tubulin to stabilize microtubules and promote microtubule assembly in the brain (Weingarten et al., 1975; Goedert and Spillantini, 2006). Tau localizes predominantly to axons and neuropil and is not observed in glia (Binder et al., 1985; Trojanowski et al., 1989).

Phosphorylation is integral to the regulation of proper tau function. However, while tau from healthy brains has a ratio of three moles of phosphate: one mole of protein, AD brains have a significantly higher ratio of 8:1 (Köpke et al., 1993). This hyperphosphorylation appears to be a major pathological change in AD, enabling tau to dissociate from microtubules. This both increases tau’s mobility and enables it to take on an oligomeric intermediary stage and the fibrillar form which aggregates in NFTs (Grundke-Iqbal et al., 1986). However, these changes do not diminish the levels of soluble cytosolic tau. An overall eightfold increase in hyperphosphorylated tau was reported, suggesting dramatically increased tau production during the course of AD (Khatoon et al., 1992). Furthermore, hyperphosphorylated (but not fibrillar) tau inhibits rather than promotes microtubule assembly by sequestering normal brain tau and microtubule-associated protein 2 (MAP2; Alonso et al., 1994, 2006; Li et al., 2007). This suggests that loss-of-function may be a major pathological process in AD, dramatically reducing the brain’s capacity for intracellular transport organelles and cell division.

NFTs spread in an anatomically orderly manner throughout the brain over the course of AD, typically projecting in an anterograde direction from the hippocampus and associated regions (Braak and Braak, 1997; Delacourte et al., 2002; Lace et al., 2009). The majority of the tau fibers which comprise NFTs are coiled helical structures referred to as paired helical filaments (PHFs), although straight filaments also occur (Braak et al., 1986). NFTs emerge in the entorhinal cortex, subiculum of the hippocampal formation and the amygdala early in the disease process. The number of tangles in the neocortex has been positively correlated with disease severity, accumulating in a consistent pattern reflecting the vulnerability of specific areas to AD pathology (Arriagada et al., 1992). However, the neuronal loss does exceed tangle formation by a significant margin, suggesting that it is not the sole contributing factor (Gómez-Isla et al., 1997).

A growing body of evidence suggests that the pre-fibrillar aggregates composed of the oligomeric form of tau have a more significant toxic role, with NFTs being unnecessary for pathology (reviewed in Shafiei et al., 2017). In fact, a transgenic mouse model of human tau demonstrated increased survival in aggregation-prone tau (d’Orange et al., 2018). This suggests a neuroprotective role in which sequestration of neurotoxic oligomeric tau mitigates its damaging effects.

### Parkinson’s Disease (PD)

PD manifests as a progressive reduction in conscious muscle control, leading to trembling, stiffness, slowness of movement and a loss of fine motor control. This is caused by selective, progressive degeneration of dopamine-producing neurons in the substantia nigra (Trétiakoff, 1919). Most cases of PD are idiopathic, however, mutations in several genes including the SNCA and parkin genes have been linked with familial forms of the disease.

A current major hypothesis suggests that aggregates develop in the brain stem and anterior olfactory structures several years before the involvement of the substantia nigra. They then propagate along the long unmyelinated axon pathways from the olfactory system and gut, leading to disruption of smell, vagal nerve function and sleep (Braak et al., 2004; Hawkes et al., 2007). However, there have been some observations of some variance in susceptibility of brain regions, temporal order and anatomical distribution, resulting in some opposition to this theory (Burke et al., 2008).

The signature lesions of PD are two types of aggregates in the cytoplasm of dopaminergic neurons; Lewy Bodies (LBs) and Lewy Neurites (LNs). Amyloid forms of ubiquitinated and hyperphosphorylated α-synuclein are the most abundant protein in LBs and LNs (Uversky, 2007; Braak and Del Tredici, 2008b). These aggregates develop in the cell body and neuronal processes respectively, and form a considerable time prior to the appearance of somatomotor dysfunction (Uversky, 2007). LBs and LNs exhibit a predictable, ascending pattern of progression, suggesting axodendritic transfer between anatomically connected brain regions (Braak and Del Tredici, 2008a). LBs are significantly more abundant in sporadic PD than they are in familial PD (Kotzbauer et al., 2004).

#### α-Synuclein

α-synuclein is a small, natively unfolded presynaptic protein. While its precise physiological functions are unknown, it interacts with multiple proteins, lipids, and membranes. It has been suggested to have roles in synaptic maintenance and neurotransmitter release, especially of dopamine (Maroteaux et al., 1988; Clayton and George, 1999; Abeliovich et al., 2000). The absence of α-synuclein does not significantly impede survival but does suggest that the protein is an essential negative regulator of dopamine neurotransmission (Abeliovich et al., 2000).

The expression of α-synuclein in PD varies between brain regions. Most α-syn expression is in the cytosol of excitatory neurons in brain regions affected early in PD such as the olfactory bulb, the dorsal motor nucleus of the vagus and substantia nigra pars compacta. However, some α-synuclein has been observed in inhibitory synapses of the external plexiform layer of the olfactory bulb, the lateral and medial globus pallidus and the substantia nigra pars reticulata (Taguchi et al., 2016).

Both wild-type and pathological variants of α-synuclein form amyloid-like fibrils upon prolonged incubation in solution, although the pathological form does so at a higher rate (Conway et al., 2000). In the process of forming these fibrils, α-synuclein takes on oligomeric forms which later develop into spherical, ring-like and string-like intermediate forms, collectively known as protofibrils. These are soluble structures which gradually coalesce into insoluble fibrils, forming α-synuclein aggregates. There is evidence that these soluble oligomers are the source of neurotoxicity in PD, disrupting cellular homeostasis and mediating neuronal death (reviewed in Stefanis, 2012). However, it has been noted that even if inclusion bodies are not the main effector of PD pathology they may exert neurotoxic effects including blocking neuronal trafficking in axons and sequestering essential neuronal components.

There is evidence of a synergistic interaction between the Tau protein and α-synuclein, as co-occurrence leads to accelerated fibrillization of both proteins (Giasson et al., 2003). However, while the proteins co-occur in the same vicinity of LNs, affected neurites typically have either tau or α-synuclein, but not both (Kotzbauer et al., 2004).

### Huntington’s Disease (HD)

HD is a progressive, autosomal dominant neurodegenerative disorder characterized selective neuronal cell death, primarily in the cortex and striatum, leading to motor disturbance, cognitive loss, and psychiatric issues (Vonsattel et al., 1985). The disease typically manifests clinically in people aged 40–50, with pathology worsening over 10–20 years until death.

HD inheritance is driven by mutations in the gene encoding the huntingtin (Htt) protein. The dominant mutation of interest is the repeat expansion of a CAG trinucleotide repeat. In unaffected individuals, the number of repeats varies between 6 and 39, while in individuals with HD the number increases to 36–180 (Rubinsztein et al., 1996; Mangiarini et al., 1997). This leads to the expansion of the polyglutamine (polyQ) tract, leading to the formation of amyloid-like protein aggregates (MacDonald et al., 1993; Scherzinger et al., 1997). Most adult-onset cases have expansions ranging from 40 to 55 repeats, while expansions of 70 and above have been associated with the juvenile form of the disease (Scherzinger et al., 1997).

#### Huntingtin

The function of Htt is not well understood. However, it is essential for development, with disruption resulting in embryonic death in mice (Nasir et al., 1995). N-terminal Htt has demonstrated the ability to shuttle between the cytoplasm and nucleus in a Ran GTPase-dependent manner, interacting with the nuclear export-associated translocated promoter region (Tpr) of the nuclear pore (Cornett et al., 2005). There is evidence for the involvement of Htt in both anterograde and retrograde microtubule-based axonal trafficking, with Htt-associated protein 1 (HAP1) mediating interactions between Htt, microtubule motor proteins and their co-factors (Schulte and Littleton, 2011). These functions are disrupted by the activity of mutant Htt (mHtt), resulting in impaired vesicular and mitochondrial trafficking (Trushina et al., 2004).

Htt is expressed in various parts of the body, including the colon, liver, pancreas, testes and the entirety of the brain. Within the brain, it is focussed on the neurons of the dentate gyrus and pyramidal neurons of the hippocampal formation, cerebellar granule cell layer, cerebellar Purkinje cells and pontine nuclei. While expression does occur in glial cells, the neuronal expression is significantly more prominent (Strong et al., 1993). The protein primarily localizes with vesicles and microtubules, and may function in cytoskeletal anchoring or vesicle transport (DiFiglia et al., 1995; Hoffner et al., 2002).

The expression of the normal and mutant forms of Htt has been shown to be similar in the CNS (Trottier et al., 1995). However, neuronal loss in HD has been found to vary by location. The putamen and caudate nucleus suffer the greatest losses (64 and 57%, respectively), followed by 29–34% in telencephalic white matter and 21–29% in the cerebral cortex (de la Monte et al., 1988). Decreases in the volume of the gray matter (cortex), white matter (axonal fibers) and increases in CSF all begin several years before the onset of symptoms. This change in CSF, in particular, progressed linearly and in association with the number of polyQ repeat expansions (Squitieri et al., 2009). Loss of striatal and white matter volume has been identified as much as 15 years prior to the onset of symptoms, suggesting that preventative treatments must be initiated long before diagnoses can be effectively made (Paulsen et al., 2010).

Pure polyQ segments can be folded into several distinct fiber conformations, which confer different levels of toxicity and consequently neurodegeneration (Nekooki-Machida et al., 2009). This has been used to justify a lack of correlation and in some cases negative correlation, between deposition of Htt aggregates and observed toxicity (Arrasate et al., 2004). It has been proposed that variants with extended β-sheets resulted in modestly toxic or nontoxic effects as a result of “buried” polyQ, which prevented interaction with, and sequestration of, free endogenous proteins (Nekooki-Machida et al., 2009).

Gain of function mechanisms such as increased levels of reactive oxygen species (ROS) and direct toxicity exerted by the polyglutamine repeat expansion appears to be the core mechanism of pathological toxicity in HD (reviewed in Imarisio et al., 2008). However, this is not likely caused by aggregate formation. Cells with Htt inclusions have, in fact, demonstrated improved survival compared to those without (Arrasate et al., 2004). It may be that aggregates are a protective measure, sequestering toxic oligomers to prevent further cellular damage. A more accurate predictor of neuronal death appears to be levels of diffuse Htt, with several studies arguing that soluble oligomeric forms of polyQ-expanded Htt are the source of HD toxicity (Leitman et al., 2013; Kim et al., 2016). Htt aggregates may instead play a role in toxicity through loss of function mechanisms. While wild-type Htt inhibits excitotoxic neurodegeneration, possibly through the binding of apoptosis-mediator caspase-3 (Leavitt et al., 2006), pathological mutant forms of Htt have been associated with a reduced binding affinity for caspase-3. This can either directly lead to or increase vulnerability to cell death (Zhang et al., 2006). In addition, there is evidence of proteasome sequestration within Htt and polyQ aggregates, facilitating pathology through the inhibition of cellular clearance processes (Holmberg et al., 2004).

### Amyotrophic Lateral Sclerosis (ALS)

ALS is a neurodegenerative disorder characterized by progressive degeneration of muscle function. Symptoms typically involve a combination of lost voluntary muscle control and uncontrolled spasms, with death commonly arising from respiratory failure. In most cases, degeneration is relatively constant until death. Molecular changes have been observed to occur prior to the development of physical symptoms, suggesting that denervation is a more direct symptom of the ALS disease process than cell death (Bertrand et al., 2018).

ALS is atypical in that multiple prionoid proteins have been associated with the disorder, independent from one another. Familial ALS (fALS) has been associated with FUS and SOD1 aggregates. Sporadic ALS (sALS) is predominantly linked to aggregates of TDP-43 and T-cell-restricted intracellular antigen-1 (TIA1). In almost all studies, the accumulation of FUS and TDP-43 in cytoplasmic inclusions has been shown to be mutually exclusive (Mackenzie et al., 2010). This suggests the presence of at least two independent disease pathways.

FUS, TDP-43 and TIA1 each have a “prion-like domain” (PLD). These are low-complexity sequences found in the genetic code of RNA-binding proteins. These sequences are enriched in glycines and uncharged polar amino acids such as asparagine, glutamine, and tyrosine (Couthois et al., 2011; Hennig et al., 2015). They are in either the N- or C-terminals of the protein (the start and end of an amino acid chain respectively). Many genes that encode PLD-containing proteins are essential in mammals, typically having roles in RNA processing (March et al., 2016). Aggregates of RNA-binding proteins are involved in various stages of mRNA processing, storage, and decay. PLDs allow these proteins to “functionally aggregate,” forming higher-order assemblies and cytoplasmic foci such as P-bodies and stress granules (SGs; Gilks et al., 2004; Buchan et al., 2008; Toretsky and Wright, 2014). However, these same aggregates can cause severe cellular damage if protein quality control mechanisms are disrupted, allowing them to propagate unchecked.

#### Fused in Sarcoma (FUS)

FUS is an RNA-binding protein that shuttles between the nucleus and cytoplasm (Zinszner et al., 1997). It has functions in transcriptional regulation, RNA homeostasis and rapidly appears at sites of DNA damage, suggesting a role in DNA repair (Bertoletti et al., 1996; Kasyapa et al., 2005; Wang et al., 2013). The protein is a major component of cytoplasmic SGs and results in the co-localization of SGs to autophagosomes (Bosco et al., 2010a).

Most ALS-linked FUS mutations are clustered within the C-terminal domain. Many disrupt the nuclear import of FUS and thus promote cytoplasmic accumulation. The resultant level of cytoplasmic mislocalization has been correlated with ALS disease onset, with stronger mutations resulting in earlier disease onset and more cytoplasmic FUS (Dormann et al., 2010). Mutations in FUS have been observed in fALS and rarely sALS, accounting for 4% and 1% of cases respectively. FUS mutations result in several forms of ALS, with P525L mutations causing juvenile (the early 20s) ALS and R521C and R518K mutations causing a late-onset disease (40s–60s; Deng et al., 2014). Post-mortem tissues from patients with FUS mutations have expressed cytoplasmic aggregates containing FUS in MNs and glial cells (Kwiatkowski et al., 2009). ALS-linked variants of FUS appear to engage different RNAs than wild-type variants, which may contribute to ALS toxicity (Hoell et al., 2011).

According to a yeast model, FUS toxicity requires cytoplasmic aggregation, the presence of a prion-like N-terminal domain and binding of RNA. This suggests that toxicity may be based in the loss-of-function effects of RNA sequestration or otherwise disrupting the activities of RNA (Sun et al., 2011). Glutamine has been observed to promote the formation of toxic oligomers species, and so the high glutamine: asparagine ratio (~6:1) of the FUS PLD may contribute to the fixation of proteins into toxic forms (Halfmann et al., 2011).

#### Superoxide Dismutase 1 (SOD1)

SOD1 is one of the most common genes implicated in ALS. While there is no consensus on the proportion of ALS attributed to SOD1 mutations, it is typically held to account for between 13% and 20% of familial ALS and between 0% and 7% of sporadic ALS (Andersen, 2006; Chiò et al., 2008). It is a superoxide radical-scavenging enzyme that natively converts the superoxide anion O_2_ into ${\text{O}}_{2}^{-}$ or H_2_O_2_, and in doing so clears free radical by-products which cause oxidative stress. It is present in the cytoplasm and nuclei of all cell types.

In its native form, SOD1 is an extremely stable homodimer. However, most ALS-linked SOD1 mutations destabilize the protein. This makes it more likely to expose typically obscured hydrophobic surfaces, increasing vulnerability to partial unfolding and leading to the formation of pathological protein aggregates (Tiwari and Hayward, 2005; Nordlund and Oliveberg, 2008; Münch and Bertolotti, 2010). This is extremely atypical in prion and prionoid pathology, which usually involves a change from instability to stability.

The oxidation appears to be a requirement of SOD1 aggregation, with aggregates lacking oxidants failing to form aggregates at all. The only exception was zinc-deficient SOD1, which itself produced significantly higher levels of aggregation when oxidated (Rakhit et al., 2002). Aberrant oxidation or post-translational modification of SOD1 has also been observed to promote aggregation in *in* *vitro* models (Rotunno and Bosco, 2013).

While the pathological activities of mutant SOD1 (mSOD1) are generally considered to have their basis in gain-of-function mechanisms, there is also evidence of lost function. Notably, almost all ALS-associated SOD1 mutations result in a decrease in SOD1 enzyme activity, and SOD1 knockout models demonstrate similar outcomes to ALS (Saccon et al., 2013). These effects include increased oxidative stress, susceptibility to neuron loss following injury and progressive motor neuron degeneration (Reaume et al., 1996; Fischer et al., 2012; Shi et al., 2014).

#### TAR DNA-Binding Protein 43 (TDP-43)

TDP-43 is a highly conserved essential RNA-binding ribonucleoprotein. TDP-43 prionoid proteins express ordered, self-perpetuating aggregation transmissible from affected cells to their progeny. Their properties suggest a closer relation to yeast prions than human prion protein (PrP; Polymenidou and Cleveland, 2017).

TDP-43 is the major protein in most ALS-linked cytoplasmic inclusions (Scotter et al., 2015). Increased levels of TDP-43 mRNA have been observed in the motor neurons of both fALS and sALS patients, with TDP-43 aggregates forming in their motor cortices and spinal cords (Arai et al., 2006; Rabin et al., 2010; Qin et al., 2014). The development of TDP-43 inclusions results in increased export of TDP-43 from the nucleus to the cytoplasm, leading to a sustained decrease of nuclear TDP-43 and increased levels of stable TDP-43 mRNAs (Polymenidou and Cleveland, 2017). Upon moving into the cytoplasm, TDP-43 undergoes defective phosphorylation and conformational changes followed by ubiquitination, preventing re-entry into the nuclear compartment (Neumann et al., 2006, 2009; Ayala et al., 2008; Braak et al., 2017). Disruption of intranuclear TDP-43 expression and RNA metabolism, as well as the noxious effects of toxic TDP-43 forms, results in loss of function for affected cells (Neumann et al., 2009; Ratti and Buratti, 2016).

Mutations in TDP-43 appear to either increase aggregation propensity of TDP-43 or promote SG formation, resulting in most patients with TDP-43 mutations developing a classical ALS phenotype (Manghera et al., 2016; Polymenidou and Cleveland, 2017). Cells expressing mutant TDP-43 also form larger SGs, and are incorporated into SGs earlier, than those expressing wild-type TDP-43 (Dewey et al., 2011).

Out of more than 44 ALS-linked mutations in TDP-43, all but 3 are found in the C-terminal PLD (Da Cruz and Cleveland, 2011). Elevated expression of TDP-43 C-terminal fragments containing the PLD has resulted in increased toxicity and aggregation of cytoplasmic TDP-43 in various contexts (King et al., 2012). This suggests that the PLD plays a key role in pathological aggregate formation in ALS. However, Aggregates may also occur due to the supersaturation of TDP-43 and other proteins in MNs (Yerbury et al., 2019) which can undergo liquid-liquid phase separation in the cytoplasm, leading to the formation of phosphorylated, insoluble aggregates regardless of whether or not mutations are present in TDP-43 (Gasset-Rosa et al., 2019). This may even be a precursor to protein misfolding and self-templating protein aggregation, but there is currently no experimental data to answer this question.

#### T-Cell-Restricted Intracellular Antigen-1 (TIA1)

TIA1 is an RNA-binding protein that assembles into membrane-less organelles such as SGs. Seeded TIA1 aggregation through a PLD is a requirement of SG formation (Gilks et al., 2004). TIA1 recruits other mRNAs and proteins to SGs, including TDP-43 and FUS (Polymenidou and Cleveland, 2017). TIA1 may serve to initiate aggregation, facilitated by “scaffolding” proteins and RNA molecules (Deleault et al., 2003).

TIA1 exhibits increased mutation of its PLD in ALS patients. If these mutations are a cause of ALS, it is only in a very small minority of cases (0.5% of sALS, 2% of fALS; Mackenzie et al., 2017). A study by Mackenzie et al. (2017) studied a novel ALS/FTD family and identified the P362L mutation in the PLD of TIA1. Subsequent genetic association analyses revealed a significant increase in the burden of TIA1 PLD mutations in ALS patients relative to controls. Interestingly, the post-mortem neuropathology of five TIA1 mutation carriers clearly showed a consistent pathological signature with numerous round, hyaline, TDP-43-positive inclusions. It appears that the TIA1 mutations significantly increased the propensity of TIA1 protein to undergo phase transition. In live cells, TIA1 mutations delayed SG disassembly and promoted the accumulation of non-dynamic SGs that harbored TDP-43. Moreover, in this study the TDP-43 in SGs apparently became insoluble, impinging on its mobility. These suggest that TIA1 may have a supportive role in TDP-43 pathology, rather than functioning as a sole pathogen.

These TIA1 mutations in ALS/FTD further reinforce the intrinsic role of RNA metabolism, RNA-binding proteins, and SG dynamics in ALS/FTD pathogenesis.

## Common Prionoid Features

### Propagation

**Figure 1:**
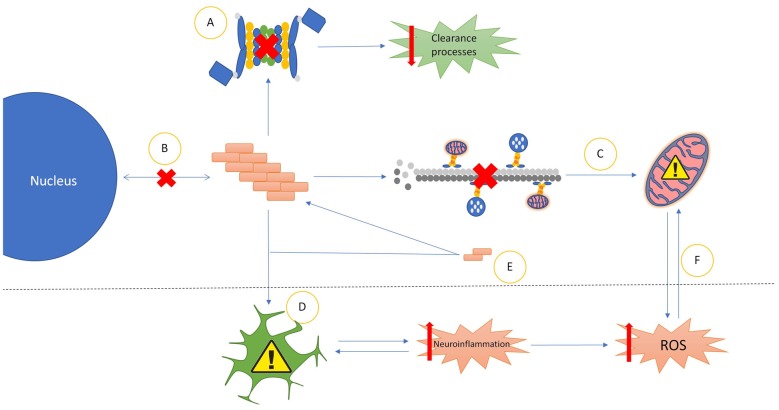
Common properties of prionoid disease pathways. **(A)** Most prionoid proteins have been associated with inhibition of the cell’s autophagic machinery, particularly the lysosomal autophagy and ubiquitin-proteasome systems. This results in inhibited cellular clearance processes, enabling greater accumulation of aggregates. **(B)** Aggregated proteins often mislocalize into aberrant cellular compartments. In amyotrophic lateral sclerosis (ALS), TAR DNA-binding protein 43 (TDP-43) and fused in sarcoma (FUS) mislocalize from the nucleus to the cytoplasm. Amyloid-β in Alzheimer’s diseases (AD), α-synuclein in Parkinson’s disease (PD) and mutant superoxide dismutase-1 (mSOD1) in ALS mislocalize into the mitochondria. In Huntington’s disease (HD), Huntingtin (Htt) mislocalizes into the nucleus. **(C)** Many Neurodegenerative Disease (ND) processes involve the disruption of microtubule-mediated transport of various cellular components, particularly mitochondria. This often results in incorrect distribution of mitochondria, enhancing mitochondrial dysfunction. **(D)** Glial cells exert various neurotoxic and neuroprotective effects. In NDs these are often either insufficient to control pathological processes or subverted to enhance pathological spread or severity. The most common mechanism of this is increased neuroinflammatory activity, leading to the production of high levels of neurotoxic reactive oxygen species (ROS). The resultant oxidative injury enhances glial activation, compounding the effects of the pathology.** (E)** Soluble oligomeric species of prionoid proteins are often present in the cytoplasm of infected cells. While typically incapable of seeding aggregates, they are recruited to aggregates in order to accelerate their growth. There is evidence that oligomeric prionoids exert neurotoxic effects. Oligomers may contribute to the spread of pathology through uptake by microglia after being exocytosed. **(F)** Dysfunction in mitochondria leads to increased production of neurotoxic ROS. The downstream effects of ROS activity can damage mitochondria, resulting in a self-reinforcing cycle.

**Figure 2:**
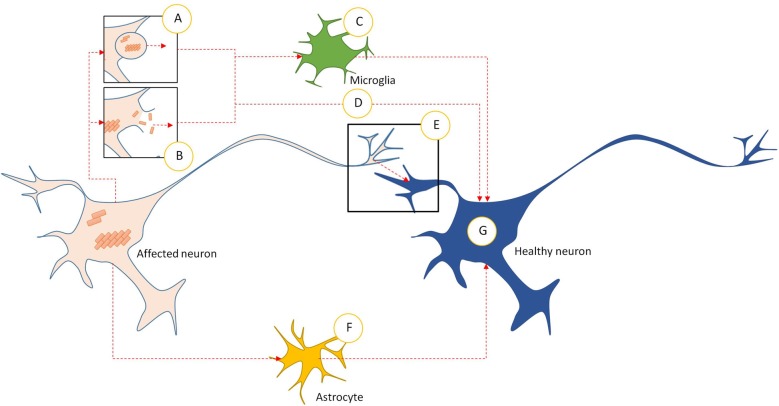
Intercellular transmission pathways in prionoid NDs. **(A)** Prionoid aggregates and oligomers are released into the extracellular space through exocytosis. This can occur in normal cells, but may be accelerated during pathology. **(B)** Cell death releases aggregates and oligomers developing within the affected neuron. Some of these are capable of persisting in the extracellular space. **(C)** Microglia phagocytose extracellular aggregates and oligomers. Many pathological processes weaken enzymatic degradation processes, allowing engulfed prionoid material to persist. Some prionoids may be released, either in vesicles or following death of the microglia, facilitating infection of neighboring cells. **(D)** Extracellular aggregates and oligomers can be taken into healthy neurons by unconfirmed processes. These include endocytosis, micropinocytosis and protein-mediated uptake. **(E)** Prionoid material can be transmitted directly across synapses. Alternatively, tunneling nanotubes may facilitate direct transmission between neurons. **(F)** Prionoid material can be internalized by astrocytes, possibly at the synapse or through tunneling nanotubules. Aggregation can progress within the astrocytes, or prionoid material can be transferred to healthy neurons through tunneling nanotubes. **(G)** Once prionoid material has entered a healthy neuron, it can seed pathological aggregation. Aggregates may seed conformational changes in natively conformed proteins, while oligomers may either accelerate the growth of seeded aggregates or infect functional amyloid aggregates.

The Prionoids function like traditional prions, in that they require both a method of seeding template-based alteration to transform natively conformed proteins into their prionoid form (Figure 1E), and a means of transmission between host neurons (Figure 2).

In AD, it was observed that injection of pathological Aβ brain extracts and homogenates into APP transgenic mice resulted in the development of associated plaques and pathology in a time- and concentration-dependent manner (Meyer-Luehmann et al., 2006). Furthermore, it was found that Aβ can be transmitted between neurons directly *via* interfaces between neurites (Nath et al., 2012). Similarly, Frost et al. (2009) demonstrated that extracellular Tau aggregates, but not monomers, can transmit a misfolded state from the outside to the inside of a cell. These induce fibrillization of intracellular full-length Tau, which is by itself capable of seeding the formation of fibrils composed of recombinant Tau monomers (Friedhoff et al., 1998). Microglia have been found to package defective tau proteins into exosomes, nanoscale vesicles secreted by all mammalian cells. While these can act to limit aggregate accumulation within cells, they can also transport the infectious proteins between adjacent brain areas, accelerating the spread of pathological proteins (Asai et al., 2015).

In PD, intracellular transmission of α-synuclein fibrils, but not monomers, can also seed the formation of LB-like aggregates, actively recruiting and converting soluble endogenous α-synuclein in the cytoplasm into a misfolded state (Luk et al., 2009). There is evidence that α-synuclein is secreted into extracellular space by atypical exocytosis processes as a normal part of the protein’s life cycle, independent of any pathological processes. However, the amount secreted is elevated in response to cellular defects associated with PD pathogenesis, such as proteasomal and mitochondrial dysfunction. Both monomeric and aggregated α-synuclein are secreted in this manner (Lee et al., 2005). The internalization of this extracellular α-synuclein may contribute to the spread of PD, as internalized material seeds aggregation of endogenous α-synuclein (Hoffmann et al., 2019). However, there is some debate as to the precise mechanism involved in this internalization. Proposed mechanisms include endocytosis, micropinocytosis, and cell surface protein-mediated uptake (reviewed in Rodriguez et al., 2018).

Large, internalized aggregates of Htt polyQ have been observed to bind to cell plasma membranes in culture, forming nuclei that selectively recruit soluble cytoplasmic proteins. These aggregates persist when cells divide, resulting in a heritable, self-sustaining seeding and fragmentation process (Ren et al., 2008). There is also experimental evidence that mHtt may be transmitted across synapses based on transgenic mouse and fly models (Pecho-Vrieseling et al., 2014; Babcock and Ganetzky, 2015). Transmitted Htt can either recruit endogenous Htt monomers and oligomers to propagate seeded aggregation (Herrera et al., 2011) or localize to the nucleus if in possession of a nuclear localization signal. Nuclear localization often leads to cell death. Short peptides with few polyQ repeats are no less toxic than longer peptides, suggesting that the pathological role of polyQ repeats may be in aggregation efficiency rather than toxicity (Yang et al., 2002).

In ALS, both FUS and TDP-43 self-replicate through the actions of their PLDs. Evidence suggests that the C-terminal domain is integral to TDP-43’s intrinsic aggregation propensity and is involved in aberrant misfolding, toxicity, recruitment into SGs and aggregate formation (Johnson et al., 2008, 2009; Dewey et al., 2011). While TDP-43 aggregate seeding appears to be mediated by the presence of an RNA scaffold (Deleault et al., 2003), FUS aggregate formation requires both the N-terminal PLD and a C-terminal RGG (glycine-arginine rich) domain. This suggests a more complex, multi-domain process than is observed in TDP-43 pathology (Gitler and Shorter, 2011; Sun et al., 2011). Fibrils of both proteins can seed aggregation *in vitro*, allowing aberrant misfolding and protein accumulation (Furukawa et al., 2011; Nonaka et al., 2013; Nomura et al., 2014).

mSOD1-positive aggregates in human patients and fALS mouse models have shown granule-coated fibrillar morphologies rich in β-sheet structures, which facilitate template-based misfolding of native SOD1 (Kato et al., 2000). In *in* *vitro* tests, preformed SOD1 aggregates and misfolded proteins have been shown to accelerate or seed aggregation of soluble SOD1 (Chattopadhyay et al., 2008; Chia et al., 2010; Eisenberg and Jucker, 2012). It appears likely that intercellular spread requires the release of aggregates into extracellular space, followed by internalization into other cells by macropinocytosis. This allows the internalized aggregates to seed aggregation of endogenous SOD1 (Münch et al., 2011). The ability to self-propagate through fragments of misfolded fibrils suggests that fibril breakage may be a key part of the self-propagation process (Lee and Kim, 2014).

### Strain-Like Phenomena

One of the most vexing aspects of prionoid pathologies for researchers is the broad range of clinical manifestations which they can produce. While broad categories of symptoms can be sketched out, each case is, for the purposes of medicine, functionally unique. Individuals vary in clinical symptoms, clinical progression, properties and behaviors of aggregates and even brain region specificity. ALS is possibly the best example of this, with multiple accepted and several controversial subtypes based on such factors as the location and age of onset of neurodegeneration and the presence or absence of cognitive symptoms (reviewed in Siddique and Siddique, 2019). Even the definitive boundaries of disorders can be somewhat unclear, as evidenced by the debate as to whether ALS and frontotemporal dementia, a cognitive disorder, share the same disease spectrum.

While there are many hypotheses for the variability of prionoid diseases, one of the most prominent is what we refer to as the “strain hypothesis.” This posits that, much like traditional diseases, prionoid disorders possess sub-species variations. However, rather than being genetic in nature, these variations exist in the conformational states of the prionoid proteins. This results in a broad range of pathological effects despite ostensibly similar aggregates structures. Studies have observed the presence of such “conformational strains” in aggregates composed of Aβ, tau, α-synuclein and TDP-43.

Aβ deposits have demonstrated a high level of heterogeneity. Pathological aggregates have been found to form dense-core plaques, diffuse deposits, cerebral amyloid angiopathies, inert deposits, and intracellular aggregates, replicating these conformations in recruited native proteins (Carlo and Stöehr, 2018). While no clear structural predominance has been identified on a population scale, within individuals the non-pathological Aβ40 isoform has a tendency for one conformation to dominate. Interestingly, pathological Aβ42 is more structurally heterogeneous and lacks a dominant species (Qiang et al., 2017). There is clear evidence for structural divisions between some pathological subspecies, with rapid-progressing AD possessing distinct Aβ42 particles that differ in size, display of N-terminal and C-terminal domains, and conformational stability (Cohen et al., 2015). Similarly, there is evidence for structural variance between familial and sporadic AD. fAD patients with mutations in the APP gene were found to test negative to the amyloid-specific PET probe Pittsburgh compound B (PiB), commonly used to detect Aβ in sAD, despite post-mortem analysis showing cerebral amyloid deposits (Scialò et al., 2019).

Studies of tau have provided even greater evidence for this, with Kaufman et al. (2016) identifying and characterizing 18 different tau strains in a cell culture model. Each of these strains resulted in different cellular pathologies when used to inoculate transgenic mice, with distinct cell types and brain regions targeted and different rates of network propagation. There is evidence that different tau fibril types cause part of this variability, resulting in different conformations when grown on fibrils of homogenous or heterogenous 3R or 4R tau isoforms (Soto and Pritzkow, 2018). However, AD has been found to show the highest level of homogeneity when compared to other tauopathies, possible due to the relative predominance of the isoform expressing a 1:1 ratio of 3R and 4R tau (Sanders et al., 2014).

Research into α-synuclein has found that different environmental factors can alter conformations, such as reduced concentrations of salts causing a change from cylindrical to twisted, ribbon-like structures (Bousset et al., 2013). Truncation of the N-terminal or C-terminal domain can likewise result in conformational differences in α-synuclein (Guo et al., 2013). Specific conformational strains can also be produced by posttranslational modification, as shown with phosphorylation of serine 129. Phosphorylated fibers demonstrated different morphology compared to wild-type fibers and exerted higher levels of toxicity. However, phosphorylation of other fibers did not lead to changes in fibril morphology, suggesting the involvement of serine 129 in suppressing toxicity (Ma et al., 2016).

We were unable to find significant evidence for conformational strains in HD, although this does not preclude the existence of such phenomena.

While there is a great deal of speculation regarding conformational subtype in ALS due to the broad range of clinical manifestations, this remains mostly conjecture. Tsuji et al. (2012) demonstrated differing patterns of expression in insoluble material extracted from ALS brain samples through immunoblotting of protease-resistant fragments from three FTLD-ALS subtypes. Nonaka et al. (2013) found that transfected cells from brains with specific histopathological subtypes templated the same properties onto wild-type TDP-43, although this was not confirmed to be based on conformational changes. A study into peptide variations also demonstrated some variability, demonstrating that seeding aggregates with different TDP-43 peptides resulted in different phosphorylated C-terminal fragments of TDP-43 and different trypsin-resistant bands. These results were interpreted as suggesting that the variability in TDP-43 peptides allowed them to induce various pathologies, ultimately functioning in a manner similar to prion strains (Shimonaka et al., 2016).

### Persistence and Disrupted Clearance of Protein Aggregates

The formation of misfolded proteins is a normal component of physiological function. As a result, cells possess innate “quality control” mechanisms that either eliminate proteins before aggregation or clear aggregates after they have formed. Schubert et al. (2000) calculated that around one-third of newly synthesized eukaryote proteins were degraded due to misfolding or improper assembly within minutes of synthesis. A growing body of evidence indicates that a core issue in prionoid pathologies is a disruption of this balance, resulting in more misfolded proteins being produced than can be degraded.

Many, but not all, prionoid aggregates are extraordinarily difficult to degrade. This is likely a result of β-sheet structures, sheets of hydrogen-bonded β-strands which are common secondary structures in pathological aggregates (Tyedmers et al., 2010).

The autophagy-lysosome pathway is the key mechanism by which cells actively degrade misfolded proteins and damaged organelles. Macroautophagy (commonly referred to as simply “autophagy”) is a key subset of this. It involves sequestering cytoplasmic materials into double-membraned autophagosome vesicles and fusing them with lysosomes, in which they undergo enzymatic degradation (Ramesh and Pandey, 2017). Several studies have been conducted into autophagy inhibition in the absence of disease-linked mutations, producing symptoms markedly similar to prionoid NDs (Hara et al., 2006; Komatsu et al., 2006; Filimonenko et al., 2007). These include loss of neurons in multiple regions of the brain, particularly cerebellar Purkinje cells and hippocampal pyramidal neurons, and resultant impairment of motor coordination and strength. The studies also observed time-dependent formation of cytoplasmic ubiquitin-positive inclusion bodies in neurons, similar in structure to prionoid pathological inclusions. Komatsu et al. (2006) noted similar proteasome function in healthy and degrading cells, proposing that autophagy had a more significant role in, and was more impaired in, prionoid pathologies.

There is growing evidence for a model of prionoid neurodegenerative disorders in which a primary pathological mechanism is destabilization of the balance between protein misfolding and clearance (Figure 1A). This enables abnormal accumulation of aggregated proteins, enabling escalating neurotoxic effects.

Impeded enzymatic degradation has been noted in both central microglia and peripheral macrophages in AD and affects the clearance of both Aβ40 and Aβ42 fibrils (Mawuenyega et al., 2010; Lai and McLaurin, 2012). Aβ accumulation in APP mutants appears to indirectly inhibit activity of the ubiquitin-proteasome system, which may lead to self-reinforcing pathological disruption of autophagy (Almeida et al., 2006). This is a possible explanation for the identification of Aβ aggregates that are able to persist more than 6 months following the cessation of pathological Aβ production (Jankowsky et al., 2005), as well as elevated Aβ levels without a concomitant increase in protein production (Selkoe, 2001).

Inhibition of Aβ and APP production in a transgenic mouse model of AD did not lead to degradation of amyloid aggregates and pools of soluble Aβ42 but did lead to a rapid decrease in the full-length APP. This resulted in significant improvements in short and long-term spatial and working memory tasks but was ineffective at recovering episodic memory and cognitive flexibility (Melnikova et al., 2013). These results suggest that several symptoms of AD depend upon the continued aggregation of misfolded proteins, while the areas which failed to recover may be affected by existing aggregates or have suffered irreparable damage from early disease processes. The latter explanation is supported by the early and severe effects of AD on areas involved in episodic memory (Gainotti et al., 1998) and cognitive flexibility (Guarino et al., 2019).

Similarly, tau pathology has been associated with decreased autophagic activity. The onset of AD has been associated with a decrease in autophagy-related gene expression, with certain tau isoforms inhibiting lysosome-dependent autophagic processes (reviewed in Hamano et al., 2018). Furthermore, NFTs composed of tau proteins are able to persist after the death of the “host” neuron, forming extracellular “ghost” tangles (Serrano-Pozo et al., 2011).

In PD, α-synuclein inclusions cannot be effectively degraded either, despite colocalizing with essential components of both the autophagic and proteasomal protein degradation pathways. They persist even after soluble α-synuclein levels have been substantially reduced, suggesting that once formed, the aggregates have an extremely high resistance to clearance.

Extracellular α-synuclein internalized by cells has been observed to impair lysosomal activity, resulting in decreased autophagosome clearance in AD patients. This effect was greatest with aggregated α-synuclein, which more persistently accumulates within recipient cells. Oligomeric intermediates proved to be susceptible to clearance (Lee et al., 2004). Interference with lysosomes, along with other protein quality control systems, has been observed to promote accumulation of α-synuclein within recipient cells, leading to the formation of inclusion bodies (Desplats et al., 2009). This indicates the potential for a self-reinforcing autophagic inhibition loop, in which internalized α-synuclein reshapes the cell’s immune environment to facilitate further aggregation and consequently, intercellular spread. Macroautophagic inhibition has been observed to contribute to cell death in aggregate-bearing cells, suggesting a possible role for loss-of-function processes in neurodegeneration (Winslow et al., 2010). Drug-mediated autophagy induction was able to mitigate this inhibitory effect, reducing α-synuclein accumulation within cells exposed to aggregates (Hoffmann et al., 2019).

Htt fragments containing pathogenic polyQ repeats have been demonstrated to nearly completely inhibit the ubiquitin-proteasome system, a major regulator of autophagy (Bence et al., 2001). This appears to be the result of proteasome sequestration within aggregates of mHtt N-terminal fragments of polyQ expansion proteins, suggesting a pathological role for aggregates outside of direct toxicity. Furthermore, polyQ repeats are themselves resistant to proteasomal degradation, further limiting cellular clearance processes (Holmberg et al., 2004). This proteasomal inhibition has been observed to double the amount of ubiquitinated aggregates, demonstrating significant growth in pathological aggregation in the absence of effective cellular clearance processes (Waelter et al., 2001).

Distinct subsets of aggregates within ALS pathology have also been termed “irreversible.” Their formation typically involves changes to secondary and tertiary structures within the protein monomers of aggregate species (Invernizzi et al., 2012; Prasad et al., 2019). This can require only a small portion of the monomer chains in the aggregate, and during this process, small proteins often undergo a significant increase in β-sheet content. Once this state has been achieved, dissociation becomes extremely difficult. However, some success has been observed with highly concentrated chemical denaturants and high pressures (Amin et al., 2014).

Depletion of healthy TDP-43 has been shown to inhibit expression of the major autophagy component Atg7, likely through destabilization of the Atg7 mRNA. This leads to the impairment of autophagy and facilitates the accumulation of polyubiquitinated proteins (Bose et al., 2011). As such, the prevention of regular TDP-43 function due to disease-associated mutation and the resultant loss of function due to aggregation may contribute to impaired cellular clearance, and thus the spread of pathology.

Mutations in FUS inhibit downstream autophagic activity by reducing the number of omegasomes, a precursor to autophagosomes (Soo et al., 2015). They have also been associated with the accumulation of autophagy substrate p62, which is upregulated when autophagy is inhibited (Mathew et al., 2009). However, p62 itself inhibits the clearance of ubiquitinated proteins, further compromising the ubiquitin-proteasome system (Korolchuk et al., 2009). It also leads to more direct toxic effects, with accumulation leading to aberrant oxidative stress responses and the formation of SGs (Thomas et al., 2013).

### Autophagy Impairment With Aging

The incidence of most NDs increases with age. Concomitantly, the ability of the body to eliminate misfolded proteins declines with age. This decline has been observed in control of the proteostatic network, which maintains proteostasis and the elimination of dysfunctional proteins, as well as many component processes of autophagy, such as autophagosome induction and fusion with lysosomes (Donati et al., 2001; Massey et al., 2006). Aging also dampens the ability of microglia to respond to stimuli such as α-synuclein (Bliederhaeuser et al., 2016). Studies of mSOD1 in ALS demonstrated that overexpression of various ALS-linked mutants did not lead to the formation of aggregate deposits (Johnston et al., 2000; Münch et al., 2011). This has led to the proposal of a “latent development” theory, in which many aggregation-promoting mutations are active significantly before symptom onset, with pathological effects held in check by cellular control processes until age-related dysfunction tips the scales. Aging microglia in AD have been observed to become dysfunctional and exhibit decreasing neuroprotective capabilities such as binding and degrading Aβ. However, they retain their ability to produce pro-inflammatory cytokines, allowing a progressive increase in net neurotoxicity (Borchelt et al., 1997; Jankowsky et al., 2001).

The shortening of telomeres may be a factor in this age-related dysfunction. Telomeres modulate DNA stability and shorten through cell replication in the process of aging (Blackburn, 2000). Shortening telomeres are known to be associated with both accelerated synuclein pathology and impaired microglial response (Scheffold et al., 2016). The average length of telomeres is shorter (Forero et al., 2016) and the rate of telomere shortening is faster in neurons, microglia and T-cells from AD patients than in healthy controls, suggesting the involvement of pathological mechanisms (Panossian et al., 2003; Flanary et al., 2007; Liu et al., 2016). Similar findings in ALS models (De Felice et al., 2014; Linkus et al., 2016) suggest that microglial telomere shortening may influence the onset of symptoms in various NDs.

### Protein Mislocalization

Aberrant protein localization is present in many prionoid pathologies. It is typically the result of mutation or alteration of the organelle transport system, either through cargo proteins, transport receptors or general deregulation (Hung and Link, 2011; Figure 1B). This can result in pathological effects through either loss of function, in which aberrant localization prevents activity despite maintaining intrinsic function, or toxic gain of function as the protein’s typical activity becomes harmful in an aberrant location.

Studies of Aβ have demonstrated abnormal HSP60-mediated translocation of APP to the mitochondria, resulting in increased levels of mitochondrial Aβ. They also observed mislocalization of these mitochondria from axons and dendrites to the soma, and resultant disruption of mitochondrial function (Iijima-Ando et al., 2009; Walls et al., 2012). Following this, Aβ has been reported to impair mitochondrial transport without affecting either mitochondrial function or the cytoskeleton in hippocampal neurons (Rui et al., 2006). The result is mislocalized mitochondria unable to return to their native positions, possibly driving pathological toxicity.

Hoover et al. (2010) found that pseudohyperpolarized tau of the sort observed in AD mislocalized to dendrites and dendritic spines, while phosphorylation-deficient tau block mistargeting. This phosphorylation-mediated mislocalization was concluded to cause early synaptic dysfunction by suppressing AMPA-mediated synaptic responses. Disruption of postsynaptic targeting and anchoring of glutamate receptors have both been proposed as possible mechanisms (Hoover et al., 2010; Miller et al., 2014).

In HD, N-terminal Htt has demonstrated the ability to shuttle between the cytoplasm and nucleus of cells. The exact mechanism of this shuttling is inconclusive, with possible mechanisms including a nuclear export sequence in Htt and interaction with the nuclear export-associated translocated promoter region (Tpr) of the nuclear pore. Disruption of both systems in HD has been demonstrated to increase nuclear Htt accumulation (Cornett et al., 2005; Zheng et al., 2013). The amphipathic α-helical membrane-binding domain appears to be required for effective transport between the nucleus and cytoplasm, enabling targeting of vesicles and the endoplasmic reticulum. When disrupted by point mutation, nuclear accumulation and toxicity of mHtt both increased significantly (Atwal et al., 2007).

Mouse models of SOD1 have established that subjects expressing wild-type SOD1 localize to both the cytoplasm and nuclei, while mSOD1 were limited to the cytoplasm. This occurred independently of any mutations in the neurons or astrocytes studied, suggesting that it is a property of the protein rather than the cell (Lee et al., 2015).

Wild-type FUS predominantly resides in the nucleus. However, ALS-linked mutants have been observed to mislocalize to the cytoplasm. The level of mislocalization has been positively correlated with both the onset of ALS and the maturation status of the MNs affected (Higelin et al., 2016). mFUS linked to severe ALS recruits significantly more cytoplasmic FUS into SGs following stress or irradiation than those linked to mild ALS (Higelin et al., 2016). mFUS-expressing cells were also noted to express increased numbers of large, densely packed FUS-positive SGs along neurites (Higelin et al., 2016).

Mutant TDP-43 likewise mislocalizes from the nucleus to the cytoplasm. There is evidence of ubiquitinated TDP-43-containing inclusion bodies in ALS, and evidence of decreased levels of nuclear TDP-43 when such ubiquitinated TDP-43 inclusion bodies are present (Neumann et al., 2006; Geser et al., 2008). Mutant-specific TDP-43 toxicity has been associated with higher levels of cytoplasmic TDP-43 mislocalization, serving as a strong predictor of neuronal death. However, there is evidence that inclusion bodies are not necessary for toxicity, and that their presence is entirely independent of cell death (Barmada et al., 2010). This indicates that the formation of inclusions may be only a method of limiting TDP-43 translocation, rather than a direct effector of pathology.

A study by Archbold et al. (2018) into inhibition and overexpression of nuclear exporters in ALS identified several redundant nuclear export pathways. They observed that inhibition of exportin-1 (XPO1) and depletion of various exportin levels failed to significantly increase levels of nuclear TDP-43, while overexpression of exporters increased nuclear export. It may be that nuclear export is predominantly independent of active export mechanisms. A recent study demonstrated that export is size-dependent, and as a result proposed that export mechanisms are predominantly driven by passive diffusion (Pinarbasi et al., 2018). They argue that the limited effects of some exporter proteins are due to shuttling of secondary components of SGs; supplementing rather than driving the formation of inclusion bodies.

### Intracellular Transport Dysfunction

A common factor in NDs is the disruption of intracellular transport systems, typically through interference of microtubule-mediated transport (Figure 1C). This contributes to the loss-of-function and mislocalization pathologies, particularly with regards to mitochondria. Mitochondrial function is reliant on extensive intracellular transport to meet the cell’s energy needs, especially with regards to the maintenance of synapses.

AD is possibly the most involved ND in intracellular transport dysfunction due to the involvement of the MAP tau. This leads to both loss-of-function due to dissociation from microtubules and a gain-of-function inhibition of microtubule assembly in its hyperphosphorylated form (Grundke-Iqbal et al., 1986; Alonso et al., 1994, 2006; Li et al., 2007). There is evidence that this inhibitory activity is the result of tau binding to the “tracks” of the microtubules, slowing anterograde transport (Ebneth et al., 1998). Aβ is likewise involved in pathological AD processes. Exposure of cultured hippocampal neurons to soluble Aβ was shown to significantly impair transport of mitochondria along axons in both the retrograde and anterograde directions (Wang et al., 2010). Aβ42 specifically has demonstrated the ability to induce mitochondrial mislocalization, with fewer in the axons and dendrites and more localized to the soma (Iijima-Ando et al., 2009). This is likely to limit the cell’s ability to undergo neurotransmitter Exo- and endocytosis at its synapses, possibly contributing to functional deficits. There is some evidence of pathological synergy between Aβ and tau as well, with the presence of both Aβ and pathologically cleaved tau increasing levels of stationary mitochondria and levels of oxidative stress (Quintanilla et al., 2012).

In HD, the activity of mHtt has been shown to result in disruption of both anterograde and retrograde microtubule-based axonal trafficking of both vesicles and mitochondria. This is accompanied by the retraction of neurites. These effects have been shown to precede entry of mHtt into the nucleus, suggesting that it is an early part of pathology (Trushina et al., 2003). Pathological aggregates in the cytosol have been shown to “immobilize” mitochondria adjacent to them. This process involves the sequestration of wild-type Htt, which is important for fast axonal trafficking, as well as various trafficking motors and mitochondrial components (Trushina et al., 2004; Chang et al., 2006).

Several factors in PD contribute to transport dysfunction. The parkin gene, one of the major sites of mutations in familial PD, is a microtubule-stabilizing protein, and thus a logical target for pathology. However, Yang et al. (2005) found no effect of PD-linked mutations on parkin’s interactions with microtubules. Parkin mutations may, however, exert indirect effects on cell stability. Healthy parkin reduces microtubule depolymerization and consequently attenuates the activity of MAP kinase (MAPK), significantly reducing certain toxic mechanisms (Ren et al., 2009). α-synuclein has also been shown to interact with the protein tubulin, inducing polymerization of purified tubulin into microtubules. However, the effects of pathological mutations on this process are inconclusive, with some studies suggesting an increase in tubulin polymerization and other impairment (reviewed in Pellegrini et al., 2017).

Research into both fALS and sALS has found a significant role of microtubule-mediated axonal transport in MN survivability. These deficits are believed to be one of the earliest events in ALS. Kinesin and dynein proteins are believed to play a significant role, being directly involved in both anterograde and retrograde transport along with microtubule polymers (reviewed in Burk and Pasterkamp, 2019). Mutations in FUS are particularly involved in the activity of the kinesin family of proteins. Recruitment of the kinesin-1 (KIF1) mRNA and protein within FUS inclusions mediates the mislocalization of specific RNAs from axons. This leads to a loss of detyrosinated glutamate microtubules, and consequently issues with RNA localization. The mechanism of effect appears to not be related to microtubule stability, but rather through targeting the tubulin carboxypeptidase enzyme onto specific microtubules (Yasuda et al., 2017). SOD1 pathology appears more strongly associated with dynein, as MNs from SOD1^G93A^ mice display defective dynein-mediated retrograde axonal transport from the embryonic stage (Kieran et al., 2005). There is also evidence for the involvement of the anterograde transport pathway, as studied in a transgenic squid axoplasm model. Activation of p38 MAPK and the consequent phosphorylation of KIF-1, inhibiting transport along microtubules, were implicated in this process (Bosco et al., 2010b). In addition, ALS mSOD1 has been shown to reduce levels of the mitochondrial membrane protein mitochondrial Rho GTPase 1 (Miro1), a master regulator of mitochondrial axonal transport. This results in further inhibition of anterograde axonal transport of mitochondria (Moller et al., 2017). TDP-43 models have demonstrated smaller growth cones and shorter axons than controls, with impaired axonal transport and cytoskeletal disruptions (Baskaran et al., 2018). TDP-43 knockdown and mislocalization have been shown to result in decreased expression of microtubule regulator stathmin-2, which is necessary for normal axonal growth and regeneration (Klim et al., 2019). This may be the result of a polyadenylation site uncovered as a result of altered splicing during TDP-43 deficiency, resulting in a truncated, non-functional mRNA (Melamed et al., 2019). Post-translational stabilization of stathmin-2 has been shown to restore axonal regenerative capabilities (Klim et al., 2019; Melamed et al., 2019).

### Failing Defense Mechanisms Propagate Disease

Another factor which can influence the precarious balance within prionoid pathologies is the subversion of defense mechanisms intended to prevent or slow the diseases’ spread. This has been observed in multiple prionoid disorders. Many of these systems involve the ejection of pathological material from stressed cells or transport between regions. These processes can only remain beneficial as long as the proteins, fibrils, and aggregates can be effectively cleared after being removed from already affected cells. Failure to do so serves as another symptom of the loss of autophagic balance within the CNS in prionoid pathologies.

Reactive astrocytes may propagate Aβ pathology by producing a secretase required for Aβ production (BACE1; Rossner et al., 2005). As Aβ is an upstream activator of astrocytes (Lian et al., 2016), this may result in a cycle in which astrocytes become activated by neuronal Aβ pathology, only to themselves increase the production of Aβ.

In PD, microglia are responsible for phagocytotic α-synuclein clearance (Lee et al., 2008). However, the microglial activation required to initiate this process has been shown to also lead to inflammatory signaling and production of ROS (Zhang et al., 2005; Jin et al., 2007). This, in turn, facilitates the oxidization of α-synuclein in nearby neurons, compounding gain-of-function α-synuclein pathology (Shavali et al., 2006).

The release of α-synuclein fibers into extracellular space through exocytosis is accelerated under conditions that increase levels of α-synuclein misfolding, such as mitochondrial and proteasomal dysfunctions (Lee et al., 2005). In adaptive situations, this may serve to limit intracellular aggregation and facilitate extracellular phagocytic clearance processes. However, when the body is incapable of effectively clearing these extracellular proteins as a result of aging or other factors, this process facilitates the spread of infectious fibrils to previously healthy neuronal and glial cells (Cuervo et al., 2004). Once internalized by astrocytes, α-synuclein pathology can be transmitted between them through tunneling nanotubes (Rostami et al., 2017). This interaction with α-synuclein oligomers greatly increases opportunities for glial and neuronal exposure, contributing to the spread of the disease. Toll-like receptor 4 (TLR4) appears to mediate inflammatory response to astrocytic α-synuclein accumulation, increasing ROS production, levels of pro-inflammatory cytokines and microglial phagocytic activity (Fellner et al., 2013; Rannikko et al., 2015).

In ALS, a study of cell-to-cell transfer of mSOD1 in different volumes of culture media indicated that pathological SOD1 transfer is the result of cellular uptake following the expulsion of aggregates by mSOD1-containing cells (Münch et al., 2011).

### Glial Neuroprotective-Neurotoxic Equilibrium

Glial cells, particularly astrocytes and microglia, have a peculiar role in prionoid diseases, in that they exert both neurotoxic and neuroprotective effects. In the context of NDs, glia is predominantly involved in the degradation of aberrant proteins and various pro- and anti-inflammatory processes. There is a great deal of debate as to whether their neuroprotective or neurotoxic activity is more significant, although this is skewed by the tendency of pathological processes to enhance the activity of neurotoxic modalities (Figure 1D). This, in turn, prompts debate as to whether therapies should be targeted at suppressing or enhancing the activities of glial cells.

Astrocytes are specialized glial cells that respond to CNS damage through reactive astrogliosis, a complex graduated continuum of context-dependent changes regulated by various signaling events. Their effects include reversible alteration of gene expression, cell hypertrophy, and rearrangement of tissue structures into long-lasting astroglial scarring (Sofroniew and Vinters, 2010).

Microglia are the resident macrophage cells of the CNS, functioning as its first and main form of immune defense. While their function is like that of peripheral macrophages, microglia have been found to be more efficient at phagocytosis than their peripheral counterparts (Jin and Yamashita, 2016). In a resting state, they actively probe the CNS for pathological changes, targeting plaques, damaged or unnecessary neurons and synapses, and infectious agents (Gehrmann et al., 1995; Luo and Chen, 2012). However, when activated they take on either the neurotoxic M1 or neuroprotective M2 phenotypes (Li and Zhang, 2016). M1 microglia release pro-inflammatory mediators such as cytokines and chemokines which inhibit phagocytosis, as well as substances such as ROS which promote oxidative stress (Dheen et al., 2007; Sorce et al., 2014; Orihuela et al., 2016). M2 microglia produce anti-inflammatory factors, clearing cellular debris through phagocytosis and releasing various protective and trophic factors (Orihuela et al., 2016; Tang and Le, 2016). However, recent evidence has suggested that microglia exist in a continuum between the M1 and M2 states, exerting various levels of toxic and protective effects. This is supported by data that suggests that the release of the beneficial components of inflammation, such as IGF-1, occurs in mSOD1 microglia in both the pre-symptomatic and end-stages of disease progression (Chiu et al., 2013).

With respect to AD, evidence suggests that astrocytes play a neuroprotective role through uptake and clearance of Aβ aggregates (Pihlaja et al., 2008, 2011). However, this neuroprotective activity is insufficient to clear plaques, as evidenced by the stability of both amyloid plaque burden and plaque size distribution throughout the progression of the disease (Serrano-Pozo et al., 2012). However, astrocytes do preferentially target diffuse Aβ deposits over larger fibrillar aggregates, and so may serve to isolate soluble, neurotoxic Aβ and thus limit collateral damage (Perez-Nievas and Serrano-Pozo, 2018). These neuroprotective activities are diminished over the course of the disease, while neurotoxic effects increase through both the loss of counteractive neurotrophic effects and toxic gain of function (reviewed in Perez-Nievas and Serrano-Pozo, 2018).

The lipid-binding protein apolipoprotein E (APOE) appears to be a necessary component in the astrocytic degradation of Aβ (Koistinaho et al., 2004). The APOEɛ4 allele is the strongest genetic risk factor of AD, with homozygous individuals experiencing an 8–12-fold higher incidence of the disease (Corder et al., 1993). This allele has been found to promote Aβ aggregation (Hyman et al., 1995), formation into soluble oligomers (Hashimoto et al., 2012) and reduces Aβ clearance (Castellano et al., 2011). It has also demonstrated a role in tau pathology, promoting tau-mediated neurodegeneration independent of Aβ pathology (Shi et al., 2017). The strong association between AD and this allele is indicative of a more neurotoxic astrocytic phenotype. Its significance as a risk factor indicates that it may be a driver of AD pathology, allowing neurotoxic elements to overcome natural defense mechanisms. In contrast, the APOEɛ2 allele exerts a significant protective influence and has been associated with a decreased incidence of AD (Corder et al., 1994). For this reason, upregulation of APOEɛ2 and/or downregulation of APOEɛ4 may serve as valuable therapeutic tools.

Activation of microglia, on the other hand, is largely neurodegenerative in AD. Activated microglia can be induced by pathological proteins such as Aβ under disease conditions, leading to increased production of cytokines and neurotoxins, including ROS, ultimately promoting neurodegeneration (Meda et al., 1995; Coraci et al., 2002).

There is also significant evidence for increased inflammatory activity in PD compared to controls (Lecours et al., 2018). In a mouse model of PD, infected astrocytes were observed to produce A53T mutant α-synuclein, leading to the induction of severe neurodegeneration (Gu et al., 2010). However, astrocytes have also shown the ability to endocytose α-synuclein released into the surrounding microenvironment by pathological lesions, sequestering it and inhibiting further infection of neurons (Lee et al., 2010). Microglia are also highly responsive to α-synuclein deposits. Olanow et al. (2019) showed that dopaminergic neurons implanted into PD patients gradually acquire α-synuclein aggregates over approximately 14 years, and this aggregation is associated with an increased presence of activated microglia. However, microglia also exhibit both neuroprotective and neurotoxic functions in response to α-synuclein and may attempt to clear the aggregates or exhibit inflammatory phenotypes (reviewed in Lecours et al., 2018).

HD-associated neurodegeneration has been positively associated with the activation of reactive astrocytes, with mild cases completely lacking reactive astrocytosis (Myers et al., 1991). One factor involved in this is the prevention of glutamate reuptake by reactive astrocytes expressing mHtt, resulting in excitotoxic injury to neurons (Shin et al., 2005). A similar mechanism has also been described in AD (Wang and Reddy, 2017), PD (Ambrosi et al., 2014) and excitotoxicity is suspected to play a role in ALS pathogenesis (King et al., 2016), although the evidence for this is less certain. However, the link between the respective prionoid proteins and glial cells is not as detailed as the other diseases reviewed here. Further research may fully describe this link for the purpose of identifying new therapeutic modalities that function across prionoid NDs by interfering with disease-progressing behaviors of microglia and astrocytes.

Microglia exposed to or expressing mHtt are abnormally reactive to stimuli that prompt an immune response (Björkqvist et al., 2008) and are more toxic to neuronal cells (Crotti et al., 2014). Reactive microglia have been observed in the neostriatum, cortex, globus pallidus and the adjoining white matter of the brains of HD subjects, but not controls. While activation occurred in all levels of pathology, accumulation was greater in cases with more severe neurodegeneration. The processes of these microglia were clearly defined even in low-grade HD, suggesting an early microglial response to changes in the neuropil and axons (Sapp et al., 2001). Transmission of Htt aggregates to microglia through phagocytic activity has been demonstrated in a fly model of HD, enabling seeded aggregation within the microglial cells. While ostensibly neuroprotective, may serve to impair microglial clearance, and excessive accumulation may facilitate transmission to healthy cells (Pearce et al., 2015).

A rat model of ALS found that selective expression of mTDP-43 in astrocytes led to non-cell-autonomous motor neuron death and consequent denervation atrophy and paralysis. It also led to the activation of astrocytes and microglia (Tong et al., 2013). Interestingly, this rat model also identified a loss of glutamate transporters in the spinal cord of affected animals, although a definitive link with excitotoxic injury was not made.

The interplay between glial cells can shift the balance towards toxic modalities. Activated microglia can cause astrocytes to become neurotoxic reactive astrocytes that produce pro-inflammatory cytokines. This results in the rapid destruction of neurons and oligodendrocytes (Liddelow et al., 2017).

There is evidence to suggest that the functions of microglia shift throughout the course of ALS. In a chimeric model with some cells expressing mSOD1, non-neuronal cells that did not express the mutant protein exerted significant neuroprotective effects, delaying neurodegeneration and extending survival (Clement et al., 2003). Similarly, mSOD1-expressing early-activated microglia exhibit higher levels of markers for the neuroprotective M2 state, while end-stage microglia displayed a shift towards the toxic M1 state (Liao et al., 2012; Tang and Le, 2016). As such, impaired microglial accumulation in early disease stages accelerates ND progression, and methods that support early microglial activity may delay prionoid pathology (El Khoury et al., 2007).

Spiller et al. (2018) proposed a biphasic model of microglial activity based on their study of a reversible model of TDP-43 ALS pathology. They observed that while mutant, aggregation-prone TDP-43 was active, there was a significant loss of motor neurons and no significant change to microglia number or activity. However, when the expression of mutant TDP-43 was suppressed, microglia expanded rapidly and specifically cleared TDP-43, allowing the subjects to recover somewhat. This raises the possibility that the constant renewal of prionoid protein aggregates from various sources (glial cells themselves included in some NDs) overwhelms microglia. This may result in a constant state of inflammatory stimulation from stressed neurons, reactive astrocytes or autocrine signals. The sequestration of cellular resources needed for this microglial response may also contribute to the lack of response, suppressing microglial proliferation until aggregation has ceased. Despite evidence from AD and PD showing that microglia can exert neuroprotective effects (Streit, 2005; Chen and Trapp, 2016; Masuch et al., 2016), none have shown this type of temporal response.

### Glia-Mediated Neurotoxicity

Nitric oxide (NO) is a highly diffusible, reactive molecule produced by the nitric oxide synthase (NOS) enzyme. At low concentrations, it is involved in the regulation of metabolic energy levels, neurotransmission, and vasodilation (Thomas et al., 2001). The anionic form of NO, nitroxyl (NO^−^) has also demonstrated some neuroprotective effects, downregulating excessive activity by pathology-associated extrasynaptic N-Methyl-D-aspartate receptors (NMDARs; Kim et al., 1999). The major pathological effects of NO emerge when co-expressed at high levels with the enzyme complex NADPH oxidase (NOX), which is produced predominantly by microglia. This complex produces the superoxide radical (${\text{O}}_{2}^{-}$) ROS, used for phagocytic pathogen degradation in healthy cells. However, NO and ${\text{O}}_{2}^{-}$ engage in an extremely rapid (almost diffusion-limited) chemical reaction, producing the more neurotoxic ROS peroxynitrite (ONOO^−^; Rubbo et al., 1994; Mander and Brown, 2005). ONOO^−^ causes irreversible nitration or nitrosylation of specific amino acid residues, which induces aberrant protein conformation and function and inhibition of mitochondrial respiration (Hess et al., 2005; Szabó et al., 2007; Figure 1F). Nitration of Parkin was noted to initially increase but later decrease Parkin activity, and α-synuclein nitration was found to contribute to aggregation, increasing resistance to proteolysis as well as reducing lipid binding and solubility in PD (reviewed in Steinert et al., 2010). Nitrosylated proteins have been observed to accumulate in the brains of human ND patients, but not healthy controls (Nakamura and Lipton, 2016). There is evidence that the nitrosylation reaction can result in substantial neuron death, although it is uncertain whether this is a result of this amino acid modification or more direct ROS neurotoxicity (Mander and Brown, 2005). Another cell death process also appears to exist in mSOD1 ALS, as low levels of extracellular NO in mSOD1-expressing MNs, but not non-transgenic or wild-type MNS, activated a self-reinforcing NO upregulation cycle which ended in cell death (Drechsel et al., 2012).

While synaptic activation of NMDA receptors (NMDAR) is protective, extrasynaptic NMDAR activation has been shown to trigger excessive NO production. This is potentially a key pathway in AD, as Aβ oligomers have been shown to hyper stimulate extrasynaptic NMDARs (Talantova et al., 2013; Molokanova et al., 2014).

The inducible NOS isoform (iNOS) has been strongly associated with ND. It is only expressed in astrocytes and microglia following exposure to proinflammatory cytokines and components of pathogens (Mander and Brown, 2005; Saha and Pahan, 2006). Once expressed, iNOS produces sustained high levels of NO, facilitating ROS production (Mander and Brown, 2005). The pro-inflammatory cytokines IL-1β and IFN-γ are sufficient to induce iNOS activation in astrocyte cells, while some other cytokines such as TNF-α can sensitive cells to IL-1β and IFN-γ-mediated iNOS activation (Trajkovic et al., 2001). iNOS can also be activated by some neurodegenerative toxins. Aggregated Aβ peptides have demonstrated the ability to trigger iNOS activation in primary microglia (Combs et al., 2001). NO-mediated nitration of Aβ also increases its aggregation propensity (reviewed in Kummer et al., 2011), potentially resulting in self-reinforcing pathology. There is also evidence of iNOS activation in HD and ALS, however, the exact inducer has not yet been identified in these diseases (Tabrizi et al., 2000; Barbeito et al., 2004).

## Therapeutics

Prion populations have been shown to adapt to the presence of selection pressures such as anti-prion compounds, developing heterogeneity even after biological cloning. There is evidence that this variability arises at the conformational level, resulting in quasi-species that can thrive in adverse environments. The most effective replicator dominates, allowing the acquisition of resistance even in the absence of genetic variability (Oelschlegel and Weissmann, 2013). Therapeutic drug design for prions should be carefully considered with this resistance in mind.

Since prions are pure proteins with no genetic material, intuitively one thinks of an antibody as an easy way to clear prion infection. When prion-infected cells in culture were treated with antibodies against PrP, the accumulation of prion protein was reduced (Enari et al., 2001). Similarly in mice, treatment with an antibody against prion protein slowed the onset of disease symptoms (White et al., 2003). Recently, the MRC Prion Unit in London announced the possibility of a human clinical trial of an anti-PrP antibody named PRN100 to treat sporadic Creutzfeldt-Jakob disease, but no details are available yet. Monoclonal antibody trials are also underway for AD but have also met with only limited success (Prins and Scheltens, 2013; van Dyck, 2018). There is also a monoclonal antibody undergoing clinical trial for PD, but the trial is ongoing and the efficacy data are not available yet (Jankovic et al., 2018). Beyond this, new technological approaches aimed at developing antibodies against beta-sheet rich proteins characteristic of prionoid NDs are also underway with promising early results *in vitro* (Goñi et al., 2017; Manoutcharian et al., 2017).

Apart from monoclonal antibodies, many other therapeutic approaches have been developed throughout the years, with limited clinical success (reviewed in Trevitt and Collinge, 2006; Aguzzi et al., 2018). Research suggests that the key to effective therapeutic approaches is not in degrading the highly stable aggregates already formed, but in preventing the development of further pathology. This allows the body to re-establish existent but overwhelmed protective mechanisms, both slowing pathological spread and allowing the recovery of function (Melnikova et al., 2013; Spiller et al., 2018). While some secretase inhibitors such as doxycycline have already experienced significant success in animal models of AD, no such success has been observed in human clinical trials (Molloy et al., 2013). Similarly, inhibitors of the β-secretase BACE1 enzyme which cleaves APP into its Aβ form have been able to lower Aβ levels, but not recover cognitive function (reviewed in Coimbra et al., 2018). However, research is still ongoing as to whether applications at pre-clinical stages may have a stronger positive impact (Voytyuk et al., 2018).

Another target for redressing this pathological imbalance is the enhancement of autophagic processes. Even in the absence of any prionoid proteins, a loss of autophagy can lead to neurodegeneration, suggesting a key role in pathogenesis. Autophagy-enhancing agents such as rapamycin, metformin, and resveratrol have been found to have various positive effects in PD, including increased α-synuclein clearance and reduced neuronal cell loss (reviewed in Moors et al., 2017). However, these agents are not selective, and many of the agents used are involved in other pathways such as apoptosis, cell growth, and immune responses. This could cause a wide variety of detrimental side effects, limiting clinical application. However, therapies are in development that target downstream components of the autophagy pathway, which may exhibit more targeted effects (Moors et al., 2017). Extensive studies have been made into inhibitors of mTOR, a complex which inhibits autophagy. One of the most common therapeutic agents is rapamycin, used in ALS, AD and HD treatment, although many others such as resveratrol, BECN1, and calpastatin are being trialed and modeled in various NDs (Towers and Thorburn, 2016; Mandrioli et al., 2018).

Overall, very little has been done in relation to the therapeutic modulation of glial cell activation, which may also clear protein aggregates. We know that sustained inflammation kills neurons and fails to stimulate further clearance of prionoid protein aggregates. Thus, one of the ways to enhance neuroprotection during disease progression in NDs could be by modulating inflammation and subduing the over-activation of glial cells. Our research has observed that while early glial activation is predominantly neuroprotective in nature, this shifts along a continuum towards neurotoxicity through increased inflammation and the production of toxic molecules as diseases progress. If glial activation is to be effectively used as an avenue for therapeutics, more research must be undertaken with regards to both the early identifications of pathological aggregation and determination of when in the disease process the glial response turns from positive to negative. However, even in the absence of such specific mechanistic knowledge, there are some avenues of approach which can be explored. These include targeting pathways that arrest M1 activation (Liu et al., 2014), decreasing the effects of pro-inflammatory cytokines and promoting the transition to the protective M2 phenotype (reviewed in Subramaniam and Federoff, 2017).

## Concluding Remarks

While these NDs have diverse symptoms, outcomes and prionoid behaviors, their similar mechanisms of action and propagation seem to indicate that they are all different manifestations of the same core pathology. At their cores, they are all indicative of a loss of proteostatic equilibrium. The reduction of protein quality control mechanisms and increase of pathological proteins results in a pathological cascade, producing sinks of cellular resources that eventually lead to dysfunction and death. Perhaps by identifying the common elements within these diverse conditions, a mechanism could be found to disrupt these pathologies before they can develop into the debilitating and lethal conditions they are today.

## Author Contributions

NS and MK conceived the article. CW performed most of the research, in addition NS and SB also provided leads to the research. CW produced the first draft of the article. NS and SB performed substantial editing and made contributions of their own to further develop the manuscript.

## Conflict of Interest

The authors declare that the research was conducted in the absence of any commercial or financial relationships that could be construed as a potential conflict of interest.
